# 385. Sepsis-Associated Encephalopathy and Dementia Outcome: A Retrospective Cohort Study of 30,378 Adult Sepsis Inpatient Survivors at a Tertiary Medical Center in Taiwan

**DOI:** 10.1093/ofid/ofae631.120

**Published:** 2025-01-29

**Authors:** Yu-Chao Lin, Yi-Ching Chang, Che-Chen Lin, Chin-Chi Kuo, Hsiu-Yin Chiang

**Affiliations:** China Medical University Hospital, Taichung, Taichung, Taiwan; China Medical University Hospital, Taichung, Taichung, Taiwan; China Medical University Hospital, Taichung, Taichung, Taiwan; China Medical University Hospital, Taichung, Taichung, Taiwan; Big Data Center, China Medical University Hospital, Taichung, Taiwan, Taichung, Taichung, Taiwan (Republic of China)

## Abstract

**Background:**

Sepsis-associated encephalopathy (SAE) is commonly observed in critically ill patients without identifiable causes, potentially leading to neurological sequelae. Prior studies have associated the long-term risk of dementia with sepsis or in-hospital infections, or the short-term risk of mortality with SAE, rather than the short-term dementia risk with SAE. We aimed to assess the association between SAE and the 1-year risk of dementia among sepsis survivors.

**Figure 1.**
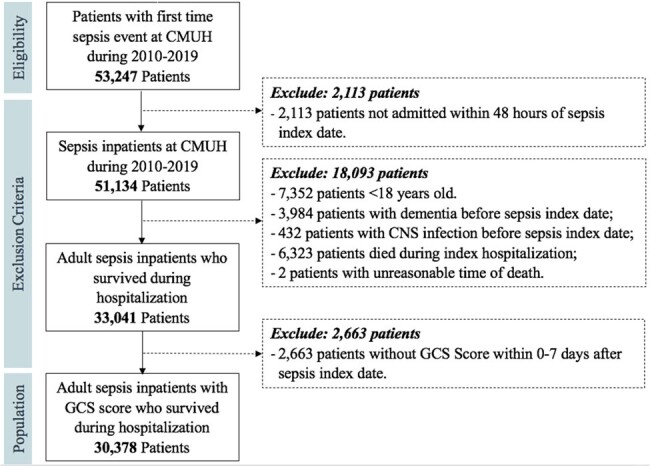
Flowchart of the selection process of adult sepsis inpatient survivors (n = 30,378).

**Methods:**

This cohort included inpatients aged ≥ 18 years with first-time sepsis during 2010-2019, no prior dementia or central nervous system (CNS) infections, Glasgow Coma Scale (GCS) measurements within 7 days of sepsis, and who survived the index hospitalization (**Figure 1**). Sepsis was identified using Sepsis-3 criteria. SAE was defined as having at least two GCS measurements of either < 7 for patients with endotracheal tubes, tracheostomies, or aphasia, or < 12 for the remaining patients within the 7 days of sepsis. Dementia at 3 months and 1 year was defined by the presence of ICD-9 and ICD-10 diagnosis codes from 7 days to 3 months, and from 3 months to 1 year post-sepsis onset, respectively. Multivariable Cox models were used, considering death as the competing risk.

**Figure:**
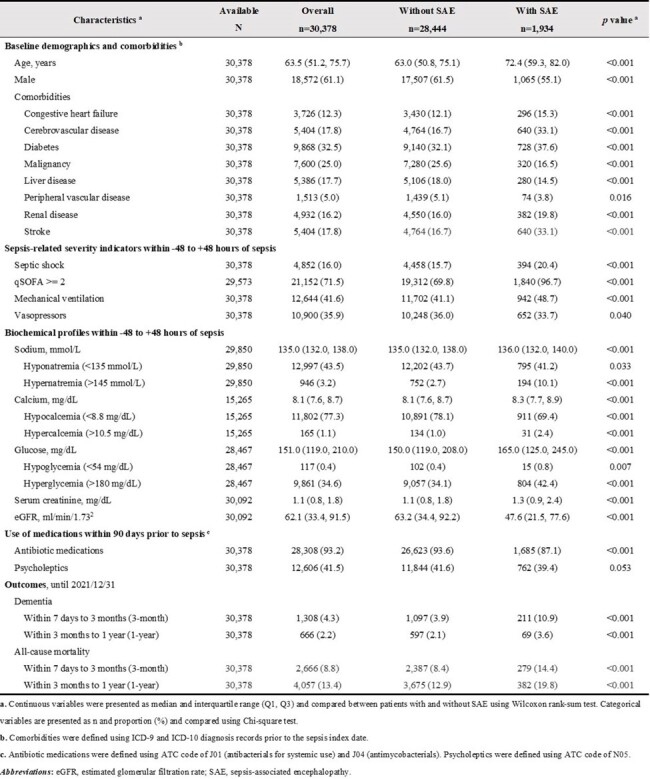

Demographic, clinical characteristics, and outcomes of adult sepsis inpatient survivors, stratified by SAE status.

**Results:**

Of 30,378 eligible sepsis inpatient survivors, 6.4% developed SAE within 7 days of sepsis onset. Patients with SAE tended to be older, female, and have more comorbidities compared to those without SAE (**Table 1**). Dementia occurred more frequently in patients with SAE than those without (10.9% vs 3.9% within 3 months; 3.6% vs 2.1% within 1 year). Similar patterns were observed in mortality rate. SAE was significantly associated with an increased risk of dementia for 3-month (adjusted hazard ratio [aHR] 1.81; 95% confidence interval [CI] 1.55-2.12) and for 1-year (aHR 1.54; 95% CI 1.18-2.01), compared to patients without SAE (**Table 2**). This association was significantly stronger among patients without chronic pulmonary disease, stroke, and with a qSOFA < 2, regarding the 3-month dementia risk (**Figure 2**).

**Figure:**
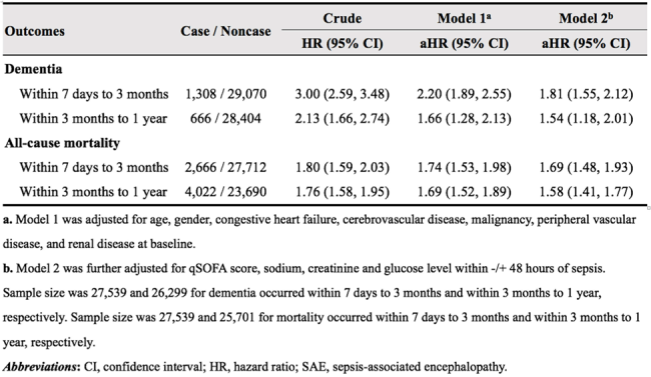

Risk of dementia and all-cause mortality associated with SAE among adult sepsis inpatient survivors.

**Conclusion:**

Determining whether SAE is a trigger for the disease itself or merely triggers more intensive dementia surveillance requires more studies with standardized SAE diagnostic criteria and post-sepsis follow-up strategies.

**Figure:**
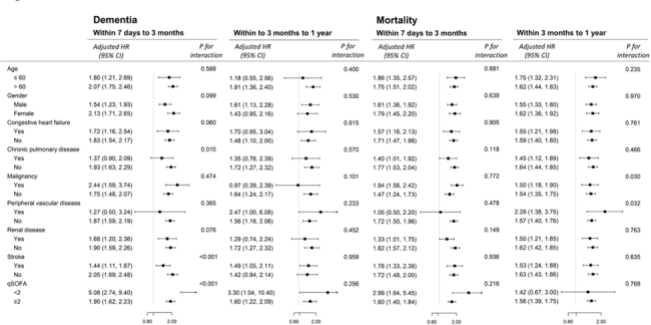

Subgroup analyses of the risk of dementia and all-cause mortality associated with SAE.

**Disclosures:**

**All Authors**: No reported disclosures

